# Healthcare leadership with political astuteness (HeLPA): a qualitative study of how service leaders understand and mediate the informal ‘power and politics’ of major health system change

**DOI:** 10.1186/s12913-018-3728-z

**Published:** 2018-12-03

**Authors:** Justin Waring, Simon Bishop, Jenelle Clarke, Mark Exworthy, Naomi J. Fulop, Jean Hartley, Angus I. G. Ramsay

**Affiliations:** 1 Nottingham University Business School, Jubilee Campus, University of Nottingham, Nottingham, NG8 1BB UK; 2 Health Service Management Centre, Park House, University of Birmingham, Birmingham, B15 2RT UK; 3 Department of Applied Health Research, University College London, 1-19 Torrington Place, Fitzrovia, London, WC1E 7HB UK; 4 Open University Business School, The Open University, Kents Hill, Milton Keynes, MK7 6AA UK

**Keywords:** Organisational politics, Political skill, Political astuteness, Leadership, System change, Qualitative, Ethnography

## Abstract

**Background:**

The implementation of strategic health system change is often complicated by the informal politics and power of health systems, such as competing interests and resistant groups. Evidence from other industries shows that strategic leaders need to be aware of and manage such ‘organisational politics’ when implementing change, which involves developing and using forms of political ‘skill’, ‘savvy’ or ‘astuteness’. The purpose of this study is to investigate the acquisition, use and contribution of political ‘astuteness’ in the implementation of strategic health system change.

**Methods:**

The qualitative study comprises four linked work packages. First, we will complete a systematic ‘review of reviews’ on the topic of political skill and astuteness, and related social science concepts, which will be used to then review the existing health services research literature to identify exemplars of political astuteness in health care systems. Second, we will carry out semi-structured biographical interviews with regional and national service leaders, and recent recipients of leadership training, to understand their acquisition and use of political astuteness. Third, we will carry out in-depth ethnographic research looking at the utilisation and contribution of political astuteness in three contemporary examples of strategic health system change. Finally, we will explore and discuss the study findings through a series of co-production workshops to inform the development and testing of new learning resources and materials for future NHS leaders.

**Discussion:**

The research will produce evidence about the relatively under-researched contribution that political skill and astuteness makes in the implementation of strategic health system change. It intends to offer new understanding of these skills and capabilities that takes greater account of the wider social, cultural organisational landscape, and offers tangible lessons and case examples for service leaders. The study will inform future learning materials and processes, and create spaces for future leaders to reflect upon their political astuteness in a constructive and development way.

**Trial registration:**

Researchregistery4020 [23rd April 2018].

## Background

### The ‘politics and power’ of implementing health system change

The implementation of strategic healthcare system change is notoriously difficult [1]. Existing research shows that a number of prominent ‘contextual’ factors shape change processes [2–7]. These factors relate, for example, to the availability and distribution of resources, incentives and opportunities, local cultures, regulatory pressures, leadership styles, communication patterns, public opposition, and professional attitudes. Such factors are increasingly interpreted as contributing to the complexity of care systems, which together make the implementation of change inherently difficult [8].

Of the many factors shown to influence the implementation change research repeatedly suggests that the local politics and power of the healthcare system can be significant; even if these are not always the primary focus of enquiry [1, 7]. One of the most well-documented examples of this ‘politics and power’ is associated with the power of healthcare professionals to resist or subvert reforms perceived as changing established ways of working [9–11]. This ‘professional power’ reflects the institutionalised authority of healthcare professions and the local strategies of professionals [11]. In other ways, these factors are commonly characterised as ‘organisational politics’ and relate to the competing interests, powerful coalitions or resistant groups that complicate strategic change [12].

When thinking about the concept of organisational politics, especially in context of public or healthcare services, it is useful to make a distinction between the more formal (big ‘P’) politics of government, politicians, policy-making and regulation, and the more informal (small ‘p’) politics of competing interests, coalitions and cliques, and resistant groups that are found in virtually all workplaces [12–16]. The informal ‘politics’ of healthcare is experienced all too often by those who work within care services, but it is often regarded by practitioners as an irrational complication, rather than an integral or constructive feature of service organisation [17]. Where existing research acknowledges the influence of informal politics, is it usually as an empirical observation and not the primary focus of enquiry.

This research is concerned with developing new evidence and understanding about the small ‘p’ politics of implementing strategic health system change, including how this is manifest and managed in different health and social care ‘arenas’. It is recognised that the formal and informal aspects of politics often interact, and local agendas are often rooted in formal statutory or sectoral differences, for example between health and social care [18]. We will consider this interaction, where relevant, but the primary focus is the influence of the informal politics of healthcare reform.

### Organisational politics and political skill

The importance of organisational politics and its influence on change processes has long been recognised in the fields of organisational sociology and management studies [12, 14, 19–21]. Management scholars such as Jeffrey Pfeffer [12] and Henry Mintzberg [20] suggest that all organisations are inherently ‘political’ with competing interests, workplace alliances, and power blocs that influence the way work happens. Importantly, this influence often occurs outside of, or alongside more formal management structures and processes. Pfeffer’s work speaks directly to the problems of implementing health services change, for example, where he argues that an emphasis on ‘top-level’ leadership and due process fails to recognise how change actually happens:

“By pretending that power and influence don’t exist, or at least shouldn’t exist, we contribute to…the almost trained and produced incapacity of anyone except the highest-level managers to take action and get things done.” [22] p.10

Although organisational politics can be seen as self-serving (Machiavellian) behaviour, a growing body of research shows it can have a constructive influence [18, 19]. For example, the competing interests of stakeholders need not result in destructive conflict, but can be a source of innovation, if effectively managed. Strategic leaders understanding of organisational politics is therefore pivotal to creating the necessary ‘receptive context’ for change [21].

Dealing with organisational politics in the workplace involves developing and using, what is often known as, ‘political skill’. These skills enable leaders to recognise, understand and mediate conflicting interests and build constructive coalitions when seeking to implement change; moreover, these skills often function alongside the more formal forms of authority used by leaders [22]. Ferris and colleagues [23–25] describe political skill as a person’s:

“…ability to effectively understand others at work, and use such knowledge to influence others to act in ways that enhances one’s personal and/or organizational objectives.” [24]

This concept has been elaborated along several dimensions to describe the characteristic features or traits of an individual’s political skill, which research suggests are positively associated with career success, team performance, and successful organisational change [21, 26]. It has been suggested, however, that the concept can overlook the more subtle forms of acumen, judgement and wisdom that help leaders recognise and respond to organisational politics [18]. Moreover, it tends to emphasise individual competencies to the neglect of wider social and cultural factors, including the importance of social acceptability, other status markers, and differences in age, gender and ethnicity [27]. More broadly, there is a lack of attention to ‘how’ political skill is used to manage organisational politics in terms of the situated and interactive practices of social actors. In recognition of these issues, the HeLPA study draws upon a modified conception of political skill, informed by Hartley’s evidence-based framework of ‘political astuteness’ [18]. This offers a broader conceptualisation of how leaders mediate organisational politics and is more open to the group and relational aspects of change. This is operationalised along the following lines:

- *Personal skills*: to exercise self-awareness and self-control;
- *Interpersonal skills*: to influence the thinking and behaviours of others, even in the absence of formal authority;
- *Reading people and situations*: to think about the dynamics that can occur when stakeholders come together, and recognising wider social systems and processes;
- *Building alignment and alliances*: promoting collaboration or alignment where there are different interest and motives;
- *Strategy direction and scanning*: having a sense of the organisation’s purpose and thinking about the long-term factors that may impact the organisation.

Political skill or astuteness can be easily seen with political leaders or diplomats, but such qualities are arguably integral to all forms and levels of organisational change. Although the concept has not been directly applied to health system change, existing research testifies to the importance of these skills amongst health services leaders. For example, several major studies describe how organisational change is conditioned by clinical leaders’ ability to understand local contexts, to balance priorities, shape local values, and mediate conflict [17, 28–32]. Recent research on the reconfiguration of acute stroke care further shows, for example, how local political factors shape the local implementation of national policy, from public pressures to competing professional interests. Significantly, this research describes how senior leaders have a pivotal role in mediating these pressures, and how the different approaches of system leaders can lead to different service outcomes [7, 33].

The HeLPA study also recognises that service leaders’ use of political astuteness is highly context specific. In particular, it is likely to vary in form, style and contribution within different political ‘arenas’. Drawing on Hartley and Bennington’s [34] analysis of political leadership, we use the concept of ‘arena’ to refer to the distinct domains where people, ideas, problems and resources come together, including physical or geographic ‘places’, as well as more dispersed and dynamic social ‘processes’. For this proposed study, a distinction is made between: i) ‘strategic’ arenas of higher-level policy formulation, priority setting and resource allocation; and ii) ‘operational’ arenas of programme management and service re-configuration.

### Acquiring and developing ‘political astuteness’

The pedagogical literature on workforce development suggests the acquisition and development of leadership skills occurs through a combination of, at least, three forms of learning [35]. First, through participation in formal education and training programmes, where abstract concepts or methods are taught in classroom or simulated environments. Second, through mentoring, coaching and action learning where learners are guided through individual and group reflection on ‘real world’ challenges [36]. Third, through experiential and reflective learning in the context of taking actions in relation to ‘real world’ situations [37]. To date, however, there has been limited research on the ways leaders acquire and develop political skill or astuteness. Research suggests that formal training and real-world experience are both important [26]. More directly, Hartley et al’s [38] research with public managers in the UK, Australia and New Zealand finds political skills are often acquired in a haphazard and sometimes painful manner. Few managers reported learning political skills through formal development courses or mentoring; rather than vast majority (88%) reported acquiring these skills through making mistakes in the workplace or through handling crises. More evidence is needed to both understand and meet the development needs of current and future leaders in the area of political astuteness [39].

In the English NHS, a number of established leadership programmes aim to enhance the capabilities of the healthcare workforce to implement strategic change. The *NHS Leadership Qualities Framework*, developed in the mid-2000s, described 15 aspects of leadership clustered around ‘personal qualities’, ‘setting direction’ and ‘delivering the service’. This recognised the importance of ‘political astuteness’ in terms of a) the capacity to understand the climate and culture of the organisation; b) knowing who are the key influencers and how to involve them; c) being attuned to national and local strategies; and d) understanding the inter-connected role of leadership. The subsequent *Healthcare Leadership Model* included nine dimensions and again highlights the need for leaders to understand the culture and politics of healthcare, including the informal chain of command. This suggests “*successful innovation involves the exercise of political astuteness*”, including the cultivation of relationships and building of coalitions amongst competing interests [31]. However, the more recent NHS framework for improvement and leadership development – *Developing People: Improving Care* – gives less explicit attention to the importance of political astuteness [40]. In various places, these capabilities are addressed in relation to ‘system leadership’ which involves building relationships and shared goals across organisational boundaries to help implement new service models. Yet, there is limited recognition of the need for service leaders to manage both the formal and informal politics of health and social care services when implementing strategic change.

Although political skill is acknowledged across these frameworks, there is little evidence about how it is best acquired or how it can contribute to effective change. Many of the attributes are poorly specified or subsumed within other behavioural competencies. Even where there is explicit reference to political astuteness, there is limited evidence upon which these qualities are based, and no explanation about how the concept has been adapted to the NHS context. With the pressing need to implement major strategic changes across the NHS, especially efforts to better integrate care services, we argue there is a need to better understand the acquisition and contribution of political astuteness to inform the design and content of new recruitment and learning resources for service leaders, and other change agents.

### Aims and objectives

The HeLPA study aims to investigate the acquisition, use and contribution of leadership with ‘political astuteness’ (PA) in the implementation of major health system change. The findings of this research will inform the co-production of materials and resources for the recruitment, training and development of current and future service leaders. The research objectives are:

1. To identify key theories and frameworks of PA within the social science literature, and apply these to recent evidence of health system change to understand how service leaders can constructively create a ‘receptive context’ for change;
2. To understand the perceptions, experiences and reported practices of service leaders, and other change agents, about their acquisition and use of PA in the implementation of health system change, taking into account differences in professional background, age, gender, ethnicity, geo-political context, and change context;
3. To understand how recent recipients of NHS leadership programmes think about, have acquired and make use of PA, to inform the development of new training resources;
4. To revise existing theoretical models of PA with reference to the wider social, cultural and relational context of health system change, and develop theoretical propositions;
5. To investigate how PA is used constructively by service leaders to create a ‘receptive context’ for implementing major health system change
6. To work with providers of NHS leadership training, NHS recruitment agencies, and PPI groups to co-produce recruitment and learning materials that support the acquisition, use, and development of PA for existing and future healthcare leaders.

## Methods/design

The proposed study is designed with four linked Work Packages (WPs) which are broadly informed by a qualitative narrative methodology, which is concerned with investigating and interpreting the experiences and accounts of social actors as relating these to wider social and cultural processes [41, 42].

### WP1: Systematic narrative reviews

Addressing Objective One, we will complete two systematic narrative reviews of the research literature to establish the theoretical and empirical foundations for subsequent qualitative enquiry. The first will produce a ‘review of reviews’ to establish the ‘state-of-the-art’ theories and frameworks on political astuteness, and related concepts. The second will apply the identified theories and frameworks to a review of the existing health service research literature to identify illustrative cases and common findings on the contribution of political skill to the implementation of organisational innovation and major service configurations.

Systematic reviews can take a number of forms, reflecting differences in the questions being asked, the problem or population under study, the types of methodological positions taken by research, and the coherence of empirical findings [43]. A preliminary scoping review shows that the relevant social science and health services research literatures are highly diverse, including different disciplinary and theoretical traditions (management, political science, public management, psychology, sociology), methodological positions (social experiments, cohort studies, qualitative case studies) and empirical evidence (surveys, interviews, observations). For this reason, the two systematic reviews will follow a narrative approach that is inclusive of the diverse literatures and aims to produce a thematic narrative synthesis of the literature.

The reviews will follow established strategies for searching and mapping the literature [44]. For both reviews, an initial selection of search terms will be produced in collaboration with the study Advisory Board and up to 20 external experts. Identified search terms will be used within electronic databases, e.g. ABI-Inform, PubMed, PsycINFO, Scopus, Google Scholar, with preliminary results reviewed according to title, keyword, location, date and journal quality (ranking/impact factor). Shortlisted papers will be subject to further review by at least two members of the research team to produce a final selection. The selected papers will be thematically reviewed and summarised.

The narrative ‘review of reviews’ on political astuteness will only include published review papers, book chapters or reports that explicitly review and synthesise the existing literature. This will systematically summarise: a) the character of political astuteness (e.g. behavioural qualities, practices, contexts, barriers/drivers, outcomes), b) its acquisition and development (e.g. workplace learning, training, etc.); c) forms of measurement and assessment (e.g. surveys and constructs); and d) outstanding research questions and propositions.

The review of the health services research literature will include publications, including include auto-biographic accounts of change produced by current or former service leaders, that directly focus on or use the terms organisational politics and political skill (and related terms identified in the initial review); in addition, a sample of exemplar studies of major service change will be included that describe instances of political skill, even where key terms are not used directly. Together, the two reviews will identify assumptions and propositions to inform the subsequent research.

### WP2: Interview study with experienced and aspirant service leaders

#### Purpose and approach

This work package will address Objectives Two, Three and Four by investigating service leaders’ experiences, perceptions and reported practices of ‘political astuteness’ in different strategic and operational ‘arenas’ of health system change. It will also investigate the views and experiences of NHS staff who have recently completed formal NHS leadership training to examine how political astuteness is developed through such training. This will involve carrying out biographical, narrative interviews [41, 45, 46] where participates are invited to give detailed reflective accounts of situations and events where political astuteness was involved in navigating the informal politics of implement strategic change; or where the lack of such astuteness derailed change; and how they learnt from these experiences.

#### Sampling

Unlike quantitative research, the purpose of sampling in qualitative research is not with measuring views or attitudes of different groups, but rather with describing and interpreting the experiences, meanings and beliefs of participants, usually in the context of their wider social and cultural field [47]. The sampling strategy for the HeLPA study acknowledges that the acquisition, use and contribution of political astuteness will vary between different strategic and operational arenas, geo-political settings, and implementation processes. It is also recognised that acquisition and use will vary in terms of an individual’s career background and length of service, clinical and non-clinical roles, as well as differences in gender, age, and ethnicity. The sampling strategy therefore aims to achieve diversity of perspectives from across different (horizontal) service settings, contexts and geographies, and at different (vertical) levels in terms of positions of strategic or operational influence, and career stage. Reflecting these considerations, a purposive sampling strategy (matrix) will be followed to select an estimated 40–45 service leaders working in different policy, professional, public and organisational settings. These include:

- NHS England clinical leads
- Leaders of recent major service reconfigurations, e.g. stroke, major trauma, cancer
- Representatives and leaders of public and patient advocacy organisations
- Local authority Executives and Directors of Services for social care
- Chief Executives of primary, secondary and tertiary care providers
- Directors of regional clinical and research networks
- Representatives of professional associations or specialist societies
- Representatives of charities and community support agencies
- Regional leads for ‘new care models’ (Vanguards)
- Leads for regional care system transformation, i.e. Sustainability and Transformation Plans, Accountable Care Organisations, or similar Integrate Care Systems

A further sample of 20 recent participants of NHS Leadership and Graduate Management training (within 2 years of completion of training) will be recruited to investigate their experiences of political astuteness and whether these skills were developed through completing leadership training.

These are indicative estimates of sampling for qualitative interviews and the actual range and number of participants is likely to vary as new or anticipated themes arise through data collection, and new opportunistic avenues for sampling are identified. Sampling will therefore be guided by the principles of saturation, with an emphasis on depth and richness of data, rather than frequency and range [47]. In grounded theory, ‘theoretical saturation’ is concerned with the extent to which inductively developed categories and concepts are adequately described and accounted for, but in other fields of qualitative research ‘thematic saturation’ it is used to suggest that data collection will continue until no new or substantive empirical themes are identified [48]. It is important to appreciate, however, that the possibility for complete saturation is unlikely, especially across a large range of service context and career levels with many unique experiences will be the focus of data collection [47]. As such, the study will look to achieve transparent and pragmatic saturation at the level of common analytical themes and theoretical constructions within and across the sampled groups, whilst accepting the inevitably of unique experiences.

#### Data collection

The preference is for face-to-face interviews to facilitate rapport and detailed understanding, which will be important when discussing the informal politics of health system change. All interviews will be digitally recorded with the informed consent of participants. Where consent is given, video recording with a small number of participants (5–10) will be made during post-interview debriefing to be used in online and educational materials. The narrative interviews will follow a topic guide to promote consistency across interviewers. Anticipated topics include:

1. Professional/career background: including leadership experiences;
2. Context of change: the significance of the reform agenda to the geo-political context;
3. Political astuteness ‘in action’: personal skills, interpersonal skills, reading people and situations, building alliance, and strategic direction;
4. Teams, groups and partners: the influence and contribution of other groups involved in or affected by the change initiative and the interpretation of the interests and goals of those groups;
5. Barrier and drivers to utilising political skill: countervailing forces; power blocs; competing interests and institutions;
6. Outcomes and impact: cases of change where political astuteness has played a part, and worked illustrations.

#### Analysis

Interviews will be transcribed verbatim for the purpose of interpretative data analysis. Analysis will examine the biographical narratives of participants [41] to understand their experience of organisational politics, and to understand how they describe using political skill to recognise and manage these factors. The analysis will involve a preliminary phase of close reading of transcripts, open coding, and identification of themes. Thematic narrative analysis will focus on the ‘stories’ or accounts produced by participants, as representative of their ‘sense-making’, their ‘positioning’ of themselves in relation to events or other actors, ‘moralising’ about the perceived norms and virtues of their environment, and ‘identity’ or how they see themselves. A key objective of the analysis is to deepen existing theories and frameworks with reference to the specific forms of political astuteness used within healthcare services.

### WP3: In-depth case studies of political astuteness ‘in action’

#### Purpose and approach

Addressing objectives two and five, this work package will produce in-depth descriptive and explanatory understanding of how political astuteness is manifest in the ‘real world’ situated practices of social actors involved in the implementation of strategic health system change. This will produce context-rich insight and address the empirical gaps that are associated with more experimental study designs. This research follows in the ethnographic tradition [49] and aims to develop a rich description [50] of the informal politics of health system change, and the use and contribution of political astuteness by different service leaders and change agents, working within and across different ‘arenas’. There are many styles of ethnographic research (e.g. realist, critical, institutional) [51], but most are concerned with direct observations of social practices and situations, and the analysis of these in relation to broader social, cultural and political institutions. Consistent with the wider study methodology, this work package adopts a narrative ethnographic approach of combining traditional methods of observation with narrative analysis to examine the storytelling and meaning making of participants in their local contexts [42]. This will involve focused observations of key meetings, project events, situations and groups interactions, illustrative of different strategic and operational arenas, combined with ‘in situ’ ethnographic interviews, and further narrative interviews with local participants.

**Fig. 1:**
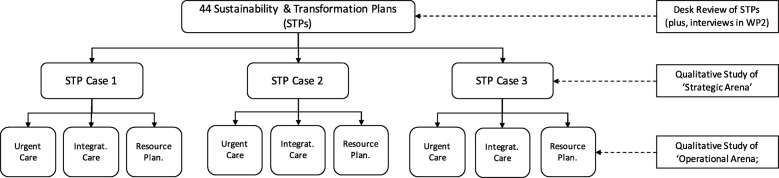
Illustration of Work Package 3 Case Study Design

The research will be carried out with three regional case studies of major health system change. Case study research aims to produce a detailed analysis of a given, exemplary case of a broader phenomenon, where the intention is depth of analysis, the elucidation of processes over time and in context, and explaining differences, rather than generalisation [52, 53]. Reflecting the idea that political astuteness will vary between and across different ‘arenas’, data collection will also focus on three internal or sub-cases. Specifically, the study makes a distinction between the ‘strategic’ arena of regional policy-making, prioritisation, resource allocation, etc., and the ‘operational’ arena of project and change management; with the expectation that forms of organisational politics and political astuteness will vary between these arenas. As such, the planned approach is to ‘zoom-in’ and narrow the focus of data collection to develop a fine-grained analysis of political astuteness ‘in action’ across these different arena (see Fig. 1).

#### The case studies

The in-depth ethnographic research will focus on three comparatives cases of the implementation of major system change within the English NHS. Specifically, it will investigate the implementation and development of strategies to create more integrated regional care systems, currently described in policies as Sustainability and Transformation Partnerships (STPs). Following the publication of *Five Year Forward View* [54], various transformation and improvement initiatives have been introduced across the NHS. These aim to realise a step-change in how services are organised and delivered at the local level, especially through better coordinating health and social care through new care models. Forty-four STPs have been developed across England to transform the delivery of care services at the local level, with changes expected to last beyond 2020. These have been developed by NHS organisations working in partnership with local authorities and other care agencies to strategically plan the future configuration of integrated care services. Reflecting current and longer term NHS priorities, the STPs cover a number of common transformation areas, including urgent care, integrated health and social care, centralisation of specialist services, illness prevention, new technologies, and resource utilisation. The on-going implementation and development of STPs is likely to see them evolve into new or additional strategic programmes for major system change, such as Accountable Care Organisations or Integrated Care Partnerships, but with the continuing goal of realising system-wide change at the regional level. This research will focus on three STPs case studies and will further compare three sub-areas or domains of transformation within each STP (see Fig. 1).

#### Sampling & Selection

A preliminary desk review of all 44 STPs proposals has identified key similarities and differences, in terms of strategic objectives and leadership. Building on this review, selection of STP case studies takes into account anticipated differences in geo-political context (population, metropolitan, rural), strategic priorities, and leadership arrangements. The research will focus on three STPs, with two selected in the English Midlands and one in London. This will enable comparison between STP based in distinct geo-political settings (London and Midlands) and also the interaction of STPs working in adjacent areas (two Midlands cases).

The case study research will be undertaken at different levels or ‘arenas’ of change. The study will, at first, focus on the overarching ‘strategic’ leadership and governance of each STP case study, before focusing on three ‘operational’ sub-cases within each STP at the ‘operational’ level of change (see Fig. 1). Thus, resulting in nine comparative sub-case studies to enable analysis of differences according to contextual factors and leaders’ use of political astuteness. Informed by the preliminary scoping review, a range of common priority areas have been identified across all STPs, and the sub-case study research will focus on transformation projects related to:

- changes to urgent care planning and provision;
- health and social care integration; and
- resource prioritisation and allocation.

These are prominent areas of change for the majority of STPs that are likely to involve different combinations of stakeholders with variable interests and sources of power. In addition, the research will focus on the key aspects of system change identified in the existing literature [1], including ‘public involvement’, ‘clinical engagement’, ‘communication and framing’ and ‘use of evidence’, with additional themes identified during WP1. Analysis of these themes is likely to provide transferable lessons to other STP priorities and major service changes in the future.

#### Data collection

The ethnographic research will be a undertaken by experienced field researchers. As outlined above, data collection will first, focus on the role of political astuteness in the ‘strategic’ arena of high level STP formulation and planning. This will involve non-participant observations of ‘high level’ STP meetings, public forums and other planning situations (estimated 5–10 meetings per STP). It will also involve semi-structured interviews, and informal in-situ interviews, with the strategic leaders and key partners. We anticipate this will include between 5 and 10 key leaders from each STP area, including Chief Executives and senior leaders from NHS Trusts, local CCG leads, Local Authorities, Healthwatch, and other regional stakeholders (estimated maximum of 30 interviews**)**. Particular consideration will be given to the role of PPI representatives in these strategic arenas, and the distinct forms of political astuteness used by these individuals and groups. As with Work Package Two, empirical and thematic saturation will guide the process of data collection [47].

Next, the research will investigate the three ‘operational’ sub-case studies for each STP. This will again involve observations of project meetings and events (estimated 5 per theme), and shadowing of service leaders (1–3 days). As with all ethnographic research, it is not possible to plan in advance all the settings to be observed or individuals to interviewed, and it is likely that many in situ ethnographic interviews will also be undertaken. It will also involve semi-structured interviews, and in-situ interviews with the service leaders and teams leading the change programmes for urgent care, health and social care integration, and resource allocation. It will also involve interviews with staff and patient representatives involved in these change areas to understand their perceptions of political astuteness. For each of the three sub-cases, we anticipate interviewing around 5–7 key people involved in project management, plus a further 5–10 staff and patient representatives. This will result in a total sample of around 135 individuals.

Data collected will occur over an 18 month period, with concurrent data collection over 6–10 months with each STP. Interviews and observations will examine, and compare, the informal political environments of change, and the lived experiences of different service leaders as they seek to formulate and implement change. It is recognised that researching the informal political environment might bring to light ethical interpersonal and organisational issues. The research team will ensure the confidences and anonymity of participants and will work to build trust and rapport with participants and researchers will be mindful and sensitive to ethical issues.

#### Analysis

All observational and in situ interview data will be recorded in field journals and electronically reproduced for data analysis. Analysis will produce three in-depth comparative case reports that will: a) identify and evidence activities that support the development and utilisation of political skill; b) understand how the political landscape varies between settings and the different skill needed; and c) revise the existing theories and frameworks of political skill and their application to the healthcare context. Interview data will be analysed as with WP2.

### WP4: Co-production of new learning activities

This final activity addresses Objective Six, and aims to use the study findings to inform the development of new learning and recruitment resources for use by the NHS and leadership education providers. Following data collection and preliminary analysis, a series of co-production workshops will be organised with the aim of developing and refining new learning materials and recruitment resources. These workshops will invite representatives of different stakeholder communities to reflect upon, deliberate, and prioritise the study themes, drawing upon their distinct experiences of, and priorities for, health system change. Workshops 1–3 will focus, in particular on the key points of learning from the study and illustrative examples of political astuteness; whilst Workshops 4 & 5 will focus on developing and appraising learning materials:

1. **Expert workshop**: a one-day workshop comprising research and practice leaders in the fields of health services research, implementation science and organisational change, to review the study findings and draw out key lessons and evidence for policy and practice;
2. **Service provider workshop**: a half-day workshop to discuss the study findings with regional and local service leaders to develop recommendations for supporting learning and change in different practice situations;
3. **PPI workshop**: a half-day workshop to review the distinct political challenges and forms of political astuteness experienced and used by PPI representatives;
4. **Educator workshop**: full-day workshop for existing leadership programmes providers to review their current curricula, to discuss understandings, models and frameworks on political skill, to consider the application of research findings to revise existing learning activities and materials;
5. **Appraisal and development workshop**: a full-day workshop with leadership providers to design and iterative learning resources, organised after pilot activities with regional leadership provider (East Midlands Leadership Academy).

Stakeholders will be facilitated to co-design new materials and resources following creative engagement methodologies, through the use of visual aids, games and role-play to devise, test-out and model potential outputs. It is anticipated the following materials and resources will be considered:

- Detailed case studies of political skill ‘in action’
- Learning exercises and scenarios based on ‘real world’ examples
- Contributions to existing and future NHS Leadership development and competency frameworks
- Workbooks for learners to explore decision-making options
- Biographies and personal testimonies of political leadership
- Online resource and social media
- Videos and audio packages which can be placed on iTunesU or other platforms
- Materials for a MOOC on “An introduction to political astuteness in healthcare” which would be of value to clinicians, managers and patient representatives.

The materials and resources will be piloted by partner universities and NHS leadership training agencies after the completion of this study. It is anticipated that this testing will be organised through the delivery of two one-day non-residential courses offered to up to 20 middle-managers and project managers (drawn from current student cohorts) in the Midlands. The pilot will assess the relevance and acceptability of the learning materials through feedback survey of participants and short telephone interview, with feedback reviewed in the final workshop to update materials.

## Discussion

This study will advance understanding on the acquisition, use and contribution of political astuteness in the implementation of strategic health system change, with a particular focus on the current implementation of regional major system changes within the English NHS. The findings will establish the ‘state-of-the-art’ theories and frameworks on political astuteness, skill and related concepts, and for the first time systematically apply these to the re-interpretation of existing health services research. Through the interview study and then ethnographic fieldwork it will develop new insight about the realities of political astuteness developing reflective biographical accounts and rich insight from ‘within’ change programmes. These findings will provide the foundations for new teaching and learning materials, and guidance for recruiters of future healthcare leaders.

A further aspiration of the study is to produce a new level of empirical insight about ‘how’ political astuteness is acquired, developed and used. The majority of studies to date have been primarily quantitative and survey-based, where a measure of political skill (independent variable) is statistically analysis in relation to various individual, team and organisational outcomes (dependent variable). Although often analytically powerful, such research does not explain how and why political skill or astuteness is used in different contexts and what to what effect. As such, the study aims to provide novel insight into the situated practices and realities of political astuteness ‘in action’. It will draw on parallel theories of strategic social change with the goal of extending existing theories [55, 56].

## Abbreviations

HeLPA: Healthcare Leadership with Political Astuteness
MOOC: Massive Online Open Course
NHS: National Health Service (England)
PA: Political Astuteness
STP: Sustainability and Transformation Partnership
WP: Work Package
